# Remembering Donald A. Enarson

**DOI:** 10.5588/ijtld.22.0048

**Published:** 2022-09-21

**Authors:** G. N. Kazi

**Affiliations:** International Union Against Tuberculosis and Lung Disease, Paris, France

With great sadness, *Public Health Action* announces the loss of its founder and first Editor-in-Chief Professor Donald (Don) Arthur Enarson on 2 June 2022, at the age of 75 years. Don, as he was lovingly called by all who knew him, had presided over the scientific programme of The Union for more than two decades until his retirement in 2011. Prior to that, he had a wonderful academic career, initially at the University of British Columbia, Vancouver, Canada, and since 1987, as a Professor of Medicine at the University of Alberta, Edmonton, Canada. He has left behind a major legacy, built on accomplishments that will continue to inspire all those working for the control of TB, lung diseases and non-communicable diseases for a long time to come. Suffice it to say that many of the public health practitioners working in both high- and low-income countries have benefited from his mentorship and support. On their behalf, we offer our thanks and our sympathy to all his family members.

Following up on the work of Dr Karel Styblo, Don was responsible for launching the five elements of the directly observed treatment short course (DOTS) strategy. In 1995, after his work was recognised by the WHO as the way forward for TB control, this was presented to all Member States of the United Nations. He subsequently monitored TB-DOTS programmes across the world, and also constituted the visiting faculty in scores of public health schools. This embedded the strengths of the strategy in the next generation of scholars, who would later lead national TB control programmes around the world and/or sit on policy making bodies of the WHO, The Union and the Stop TB Partnership. He was also a prolific writer and contributed to around 400 publications in scientific journals (including 186 original research papers), mostly on clinical, epidemiological and preventive aspects of TB and lung disease. Don always retained a holistic approach to disease control, and was an ardent advocate of applying the DOTS paradigm for the prevention and control of non-communicable disease control.^1^ The wisdom of his methodology was soon picked up by other leading figures in the fight against TB.^2^–^4^ Don regarded *Public Health Action* as an important vehicle for promoting operational research consistent with The Union’s goal of devising practical solutions for lung health.^5^ As pointed out in an Editorial in our sister journal, the *International Journal of Tuberculosis and Lung Disease*, he would have approved of the recently expanded scope of *Public Health Action* to encompass papers aimed at achieving the attainment of the United Nations Sustainable Development Goals, particularly universal health coverage.^6^

The defining characteristics of Don’s personality was the unique blend of a superior intellect and profound wisdom with extreme humility. He would never talk of his extraordinary accomplishments, but instead praise the public health colleagues he worked with, mostly in low- and middle-income countries of Asia and Africa. I first had the good fortune to meet him when we started to implement the TB-DOTS strategy in Pakistan’s southern province of Sindh. Over the period 2000–2002, he accompanied me on various visits to the hard-to-reach arid districts of Tharparkar and Umerkot. These journeys were usually quite long and gave me a rare exposure to his unique personality. We discussed everything from DOTS implementation to a much loved, old TV series, “Little House on the Prairie”, and he remarked with pride and a glitter in his eyes “I was a born in a farmhouse myself”. He was the first doctor in his family and initially took up an assignment as a medical missionary in Juba, in southern Sudan. At that time, I did not know that I would myself lead several WHO health system strengthening missions to Juba from 2007 to 2010. This demonstrates how public health intertwines our lives, sometimes in mysterious but often rewarding ways.

I mention Don’s characteristics out of gratitude and admiration for this mild-mannered gentleman who was normally unassuming and self-effacing, but forceful in pointing out that TB is not only a terrible disease, but represents social marginalisation and injustice that needs to be eliminated. Today, we are all arguing for the same things, which demonstrates his visionary nature. He continued to express these views until the very end of his long career, including in an interview in 2019 when his alma mater, the University of Alberta, bestowed on him their Distinguished Alumnus Award in recognition of the achievements of an exemplary life. His retirement, only a few years before his untimely death, allowed him to pursue his great passions – reading, stamp collecting, cooking and gardening.

His colleagues at The Union, including those of working on *Public Health Action,* will always remember with pride Don’s accomplishments, particularly in the last quarter century of his extraordinary career in public health, during which time he revolutionised TB and other disease control strategies. With hindsight, not only did Don teach us about disease control, he also taught us about compassion and humanity. Farewell, Don – we promise to carry on your mission and not let you down!
